# Engineering placenta‐like organoids containing endogenous vascular cells from human‐induced pluripotent stem cells

**DOI:** 10.1002/btm2.10390

**Published:** 2022-09-23

**Authors:** Kangli Cui, Tingwei Chen, Yujuan Zhu, Yang Shi, Yaqiong Guo, Jianhua Qin

**Affiliations:** ^1^ Division of Biotechnology, CAS Key Laboratory of SSAC Dalian Institute of Chemical Physics, Chinese Academy of Sciences Dalian China; ^2^ University of Chinese Academy of Sciences Beijing China; ^3^ Yunnan Key Laboratory of Primate Biomedical Research, Institute of Primate Translational Medicine Kunming University of Science and Technology Kunming China; ^4^ Dalian Municipal Woman and Children's Medical Center Dalian China; ^5^ Beijing Institute for Stem Cell and Regeneration Beijing China; ^6^ CAS Center for Excellence in Brain Science and Intelligence Technology, Chinese Academy of Sciences Shanghai China

**Keywords:** 3D culture, human‐induced pluripotent stem cells, in vitro model, placenta, trophoblasts, vasculature

## Abstract

The placenta is an essential organ that maintains the health of both the fetus and its mother. Understanding the development of human placenta has been hindered by the limitations of existing animal models and monolayer cell cultures. Models that can recapitulate the essential aspects of human placental multicellular components and vasculature are still lacking. Herein, we presented a new strategy to establish placenta‐like organoids with vascular‐like structures from human‐induced pluripotent stem cells in a defined three‐dimensional (3D) culture system. The resulting placenta‐like tissue resembles first‐trimester human placental development in terms of complex placental components and secretory function. The multicellular tissue was characterized by the inclusion of trophoblasts (cytotrophoblasts, syncytiotrophoblasts, extravillous trophoblasts, and other endogenous vascular cells), which were identified by immunofluorescence, flow cytometry analyses, real‐time quantitative reverse transcription polymerase chain reaction and single‐cell RNA‐seq. Moreover, the 3D tissue was able to secrete the placenta‐specific hormone human chorionic gonadotropin β (hCG‐β) and vascular endothelial growth factor A (VEGFA). The tissue responded to the inflammatory factor tumor necrosis factor‐α (TNF‐α) and VEGF receptor inhibitors. This new model system can represent the major features of placental cellular components, and function, which have not been realized in 2D monolayer cultures. The developed tissue system might open new avenues for studying normal early human placental development and its disease states.

## 1 INTRODUCTION

The placenta is the first and largest fetal organ to develop and crucial for the health of both the fetus and its mother. It has been recognized as having a lifelong impact on their long‐term well‐being.^1^ As a highly specialized extraembryonic organ, the placenta forms a maternal–fetal interface that allows for efficiently transferring nutrients and oxygen from the mother to the fetus. A successful pregnancy requires rapid growth and increased blood flow of the placenta to support the steadily increasing metabolic demands and oxygen of the growing fetus.^2^ The coordinated development of the highly vascularized placental villous tree is necessary for continued fetal growth and well‐being. Dysfunction in the placental vasculature leads to changes in the function and development of specialized placental cells called trophoblasts^3^, ^4^, ^5^ as well as common placental disorders such as pre‐eclampsia and fetal growth restriction.^6^ Pre‐eclampsia is characterized by reduced placental vascular perfusion and an altered angiogenic response, leading to maternal–fetal morbidity.^7^

Various cell lines and animal model systems have been established to study placental biology and its associated diseases. Although much of our knowledge about trophoblast lineages and placental development comes from animal models, mammals (e.g., mice and primates) display significant diversity in placental physiology, particularly in terms of degrees of trophoblast invasion into the uterine tissue and the formation of cell layers between the fetal and maternal circulation.^8^ No equivalent animal system can accurately represent human placental development. Several trophoblast cell lines, primary cells, and villous explants have been utilized as in vitro models in placental research.^1^, ^9^, ^10^ However, the cell lines (e.g., BeWo, JEG‐3) are usually generated from a variety of sources, such as primary placental tissue and malignant tissue. Each cell line has a different phenotype and cannot represent the multiple trophoblast subtypes and physiologically relevant features of in vivo human placental tissue. Although human primary trophoblast cells and villous explants are useful for investigating physiological and pathological conditions, such as embryonic development, fetal disorders, and immune diseases,^11^, ^12^ these cells are quite difficult to obtain due to the limited source of primary human tissue.

Advances in stem cell biology have facilitated the development of a new in vitro system to model human organ development and disease.^13^ In vivo, trophoblast cells perform the major functions of the placenta.^14^ After exposing to BMP4 in a two‐dimensional (2D) monolayer culture, human pluripotent stem cells (hPSCs) can be successfully differentiated into trophoblast cells, the major components of the placenta.^15^, ^16^, ^17^ In addition, human trophoblast stem cells (hTSCs) derived from first‐trimester placenta and the trophectoderm exhibit the main characteristics of first‐trimester trophoblasts.^18^ However, the hTSC lines grow as monolayers and thus do not reflect the complex morphological complexity of the early placental villi and vasculature.

Due to the self‐renewal and self‐organized property of stem cell, organoids with 3D structure are generated in recently years.^13^ Stem cell‐derived organoids can replicate the near‐physiological structure and function of native human organs, such as the intestine, retina, kidney, liver, and brain.^19^ Compared with the traditional 2D cell cultures, 3D organoids are more physiologically relevant to primary tissues in terms of cell components, architectures, and functions. They offer a pivotal system to study cellular models of human tissue and disease.^20^ Trophoblast organoids derived from first‐trimester placenta were recently shown to provide a near‐physiological model by resembling the villous placenta in vivo, and this model was used to study the maternal–fetal interactions that occur during human placentation.^21^ In addition, pluripotent stem cell‐derived trophoblast organoids provide an alternative way to study the human placenta development and disease.^22^, ^23^, ^24^ However, human placenta is a complex organ with vasculature; it contains not only the trophoblasts but also other placental components, such as vascular cells. Models that can recapture the complex cellular components, vascular‐like structure, and functional aspects of the human placenta are still scarce.

**FIGURE 1:**
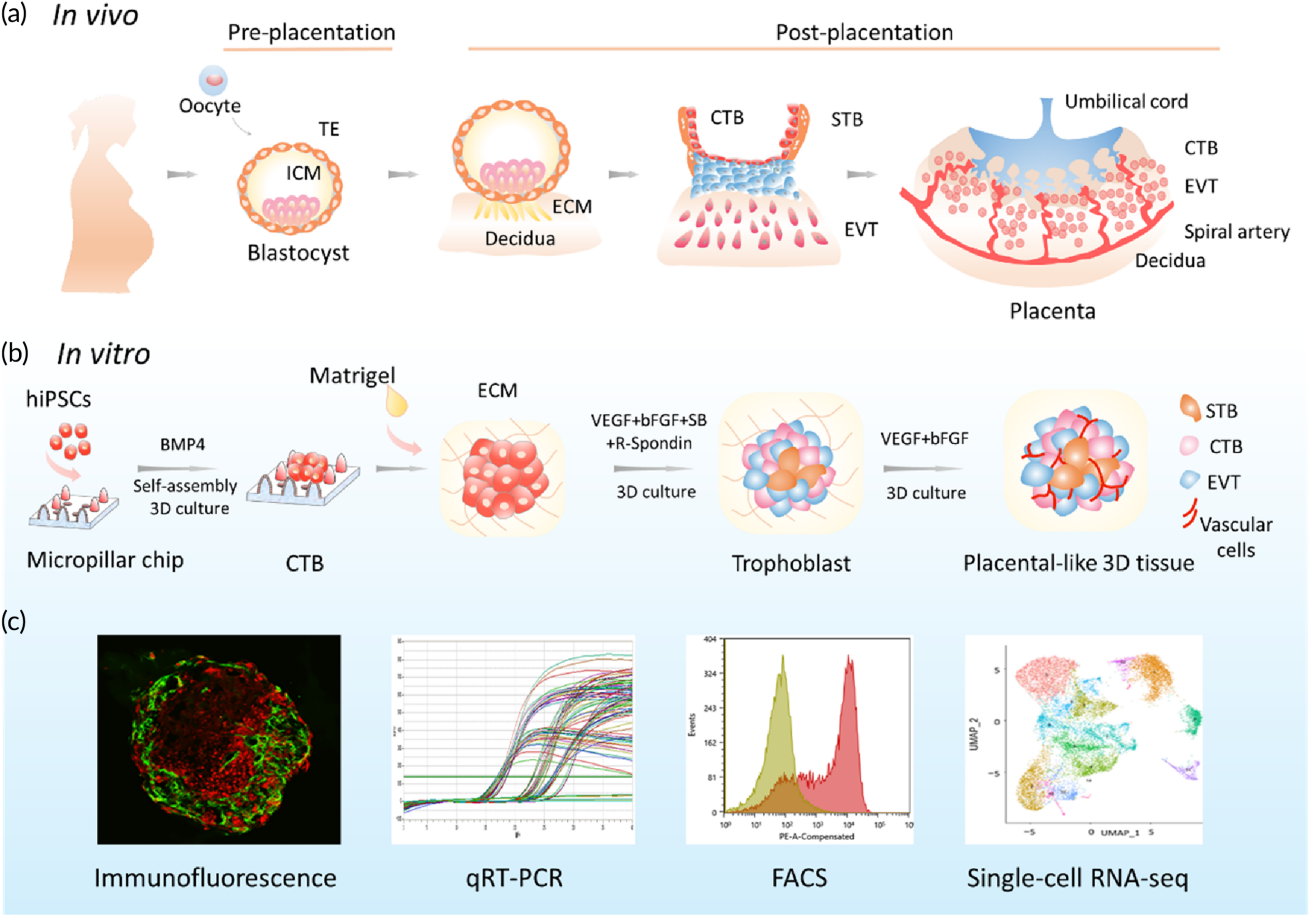
Schematic of early human placental development in vivo and in vitro. (a) Early human placental development in vivo. During pregnancy, the oocyte combines with sperm to form the zygote, thereby triggering embryogenesis. After fertilization, the blastocyst segregates into two lineages, the trophectoderm (TE), and the inner cell mass (ICM). The TE gives rise to the epithelial portion of the human placenta. As the main component of human placenta, the trophoblast is composed of three subtypes: CTBs, STBs, and EVTs. The multinucleated STBs line the outermost surface of the human placenta and subsequently form the major cellular barrier between the feus and mother. The EVTs invade into the decidua and remodel the maternal blood supply. (b) Illustration of human placental model generation in vitro. hiPSCs were seeded onto micropillar chips and treated with BMP4 to generate 3D clusters with trophoblast and mesodermal lineages under 3D culture conditions. The 3D clusters gradually grew into millimeter‐sized tissues when treated with a cohort of factors (e.g., VEGFA, bFGF, and R‐spondin 1). The formed 3D tissue contained trophoblast subtypes (CTBs, STBs, and EVTs) and vascular cells. (c) Characterization of hiPSC‐derived placenta‐like 3D tissue through immunofluorescence staining, qRT‐PCR, single‐cell RNA‐seq, and flow cytometry.

With the aim to generate the vascularized trophoblast organoids, we propose a new strategy to engineer placenta‐like organoids that contain endogenous vascular cells from human‐induced pluripotent stem cells (hiPSCs) in a defined 3D culture system in this study (Figure 1). The developed placental tissue contained trophoblast lineages, vascular lineages, and a placental villous‐like structure, resembling the key features of first‐trimester human placenta in terms of cellular components, and secretor function. Single‐cell RNA‐seq (scRNA‐seq) was used to reveal the coordinated differentiation of trophoblasts and vascular cells from hiPSCs in the 3D culture system. Such development has not been reported in 2D cultures. This 3D model system might provide a new platform for studying normal early human placental development and its disease states.

## 2 RESULTS

### 2.1 Differentiation of placenta‐like tissue from hiPSCs in a 3D culture

**FIGURE 2:**
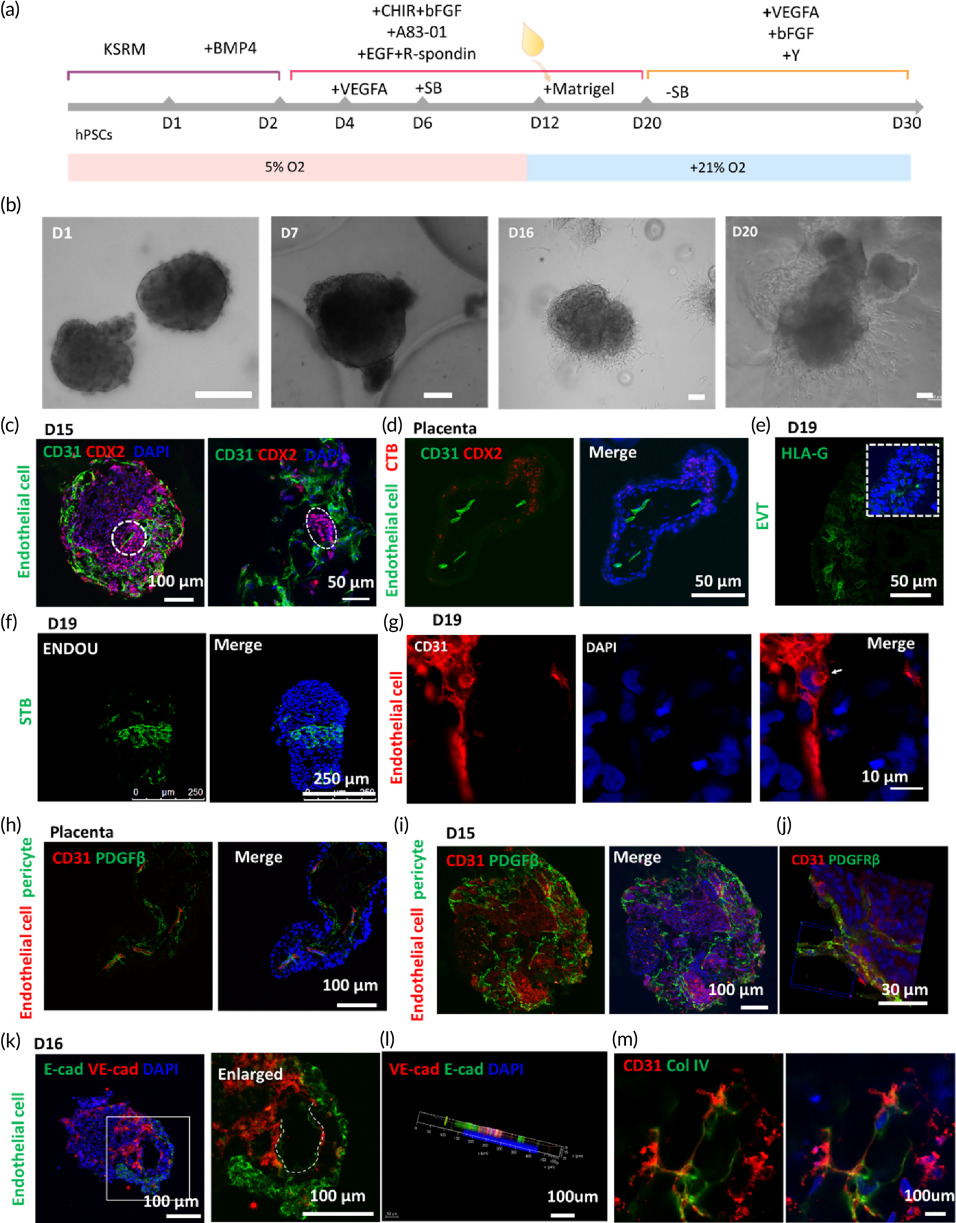
Differentiation of hiPSCs into placenta‐like tissue containing endogenous vascular cells under 3D culture conditions. (a) Schematic of the protocol used for differentiating hiPSCs into placenta‐like organoids. KSRM (KSR medium), SB (SB‐451342), CHIR (CHIR99021). The hiPSCs were exposed to a low‐oxygen environment from Days 0 to 12. At Day 12, the formed tissue was transferred to a 21% oxygen environment. (b) Representative phase contrast images of the formed 3D tissue from Day 1 to Day 20. Scale bar = 100 μm. (c and d) Confocal micrographs of the CTB marker CDX2 and the endothelial cell marker CD31 in 3D cultures at 15 days of differentiation (c) and in primary placenta (*n* = 3; 6–8 weeks gestation in first‐trimester) (d). (e–g) 3D tissue immunofluorescence analysis for the markers of EVTs (HLA‐G), STBs (ENDOU), and endothelial cells (CD31), respectively. (h and i) Confocal micrographs depicting the expression of the pericyte marker PDGFβ and the endothelial marker CD31 in the primary placenta (*n* = 3; 6–8 weeks gestation in first‐trimester) and in 3D tissue at Day 15. (j) 3D construction of the endothelial marker CD31 and pericyte marker PDGFβ in placental organoid at 19 days taken across several z‐stacks and combined into a single image by the extended focus module of the confocal microscope software. (k) Confocal micrographs showing the expression of the endothelial marker VE‐cad and the trophoblast marker E‐cad in Day 16 placenta‐like organoids. (l) 3D construction of the endothelial marker VE‐cad and the trophoblast marker E‐cad in 3D tissue. (m) Small endothelial tube marker CD31 in 3D cultures at Day 19 covered by collagen type IV (Col IV).

In vivo placental development occurs in a dynamic and complex 3D microenvironment. To achieve the conditions suited for generating placenta‐like tissue from hiPSCs, we established a multistep protocol to simultaneously induce trophoblast lineage differentiation and vascular lineage specification in a 3D culture system. BMP4 facilitates the induction of trophoblast and mesoderm lineages from hiPSCs and human embryonic stem cells in monolayer cultures.^25^, ^26^ A natural extracellular matrix (Matrigel) can contribute to the formation of 3D tissue and even organoids.^27^, ^28^ We optimized the culture conditions and investigated the feasibility of differentiating hiPSCs into placenta‐like tissues using BMP4 induction in 3D Matrigel under low oxygen conditions. The hiPSCs were seeded onto a uniformly structured micropillar chip to facilitate the generation of an array of 3D aggregates.^29^ The cellular aggregates were then treated with BMP4 (10 μM) for 2 days under low oxygen (5%) conditions (Figure 2a). After 12 days of culture, the cell aggregates were embedded into 100% Matrigel for subsequent 3D culturing. Embedding in Matrigel facilitated the rapid formation and expansion of 3D clusters within 1 week (Figure 2b). The 3D clusters were identified to contain the placenta‐specific trophoblast lineages of cytotrophoblasts (CTBs), extravillous trophoblasts (EVTs), and syncytiotrophoblasts (STBs). Flow cytometry analysis showed that the 3D clusters in the Matrigel culture exhibited higher viability compared with those cultured without Matrigel (Figure S1). Because the complicated components of the culture medium can affect the differentiation of 3D cultures, we further optimized the culture medium conditions as shown in Figure S2.

Under the optimized conditions, we examined the trophoblast‐specific cell types in the 3D clusters using immunofluorescence analysis. The results revealed that the 3D cultures contained CDX2^+^ CTBs (Figure 2c) and HLA‐G^+^ EVTs and ENDOU^+^ STBs were also present (Figures 2e–f). We also detected the endogenous vascular cells CD31^+^/E‐cadherin/VE‐cadherin endothelial cells and PDGFβ^+^ pericytes (Figure 2g,i–l). CD31^+^ endothelial cells, CDX2^+^ CTBs, and PDGFβ^+^ pericytes in primary placenta are shown in Figure 2d,h. In addition, vessel‐like structures were observed within the 3D cultures, enveloped by basement membrane collagen type IV (Figure 2m), which has not been previously reported in 2D cultures or stem cell‐derived models.

### 2.2 Single‐cell transcriptome atlas of placenta‐like tissues derived from hiPSCs

**FIGURE 3:**
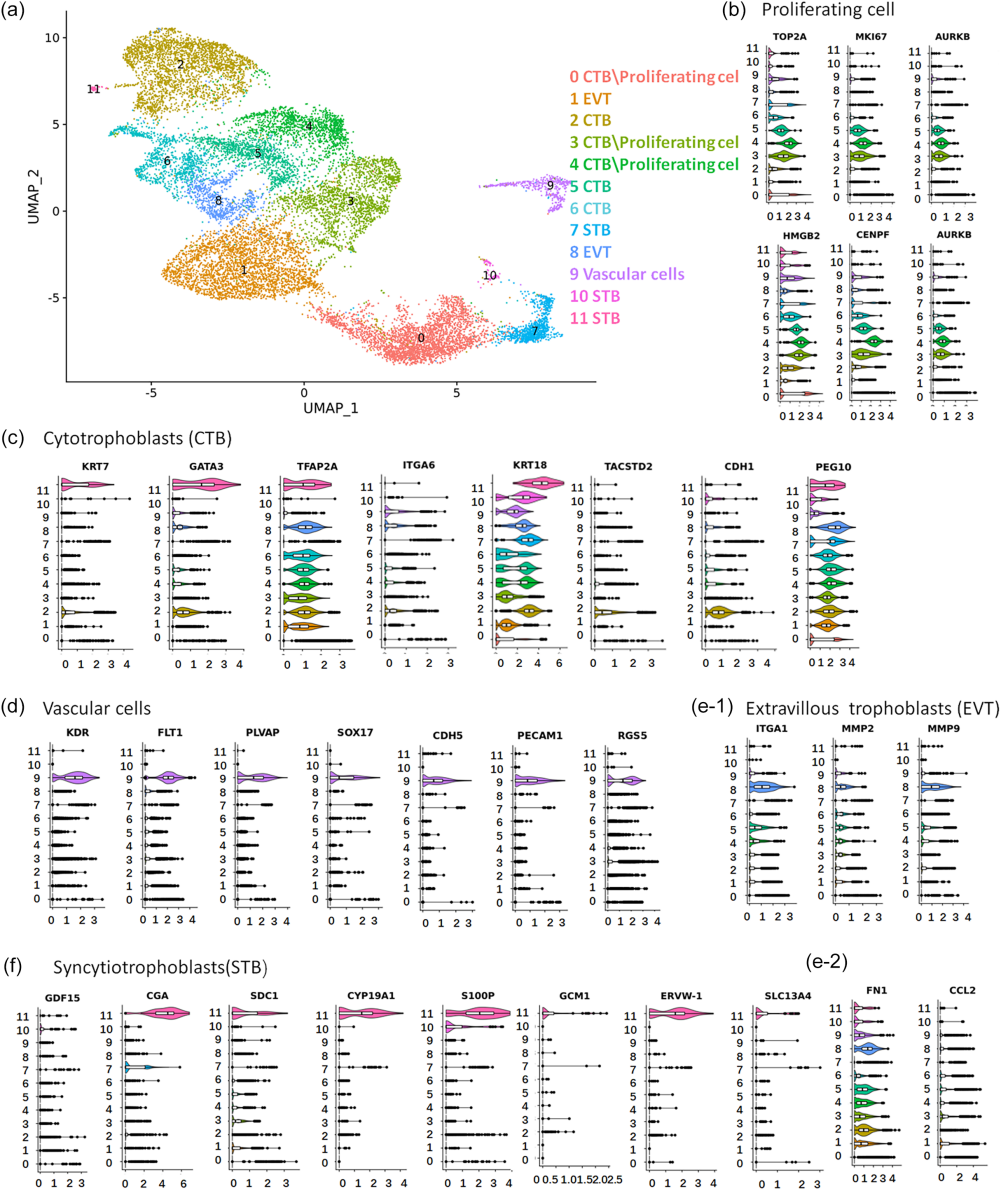
Single‐cell transcriptome atlas of the 3D tissue formed from hiPSCs. (a) UMAP plot displaying 16,311 cells at Days 9 and 24. Unsupervised clustering identified 12 clusters, which were labeled by different colors. These clusters were clustered into five cell types based on the expression of different markers. (b–f) Violin plots demonstrated the expression plot of cell‐specific genes among different clusters in the placental tissues, including proliferating cells, STBs, vascular cells, CTBs, and EVTs.

To comprehensively investigate the differentiation lineages of the placenta‐like tissue, the samples underwent scRNA‐seq, which was performed by 10x Genomics. We obtained 6507 and 9804 high‐quality scRNA‐seq profiles from the samples after 9 and 24 days of differentiation (D9 and D24), respectively. After rigorous filtering, 12 transcriptionally distinct clusters were generated by unsupervised clustering of the entire pooled dataset of 16,311 cells. These clusters were illustrated by uniform manifold approximation and projection (UMAP) using the Seurat clustering method (Figure 3a). Differential gene expression analysis was guided by established placenta‐specific markers^30^, ^31^, ^32^, ^33^ and indicated that these cell clusters were CTBs, STBs, EVTs, proliferating cells, and vascular cells (Figure 3a). The violin plots illustrate the expression of established cell‐specific markers across different clusters (Figures 3b–f).

According to published databases,^27^ the proliferating cells were marked by the expression of proliferation markers HIST1H4C, TOP2A, MKI67, CENPF, HMGB2, and AURKB. Clusters 0, 3, and 4 were identified as proliferating cells (Figure 3b). For the MKI67 marks the proliferating cell, we thus defined these proliferating cells as CTBs (Clusters 0, 3, and 4). Clusters 2, 5, and 6 were defined CTBs and were characterized by the expression of KRT7, GATA3, TFAP2A, ITGA6, KRT18, TACSTD2, CDH1, and PEG10 (Figure 3c). Vascular cells (including endothelial cells and pericyte) were identified by the expression of the canonical markers SOX17, CDH5, FLT1, PECAM1, KDR, RGS5, and PLVAP. Cluster 9 was defined as vascular cells (Figure 3d). The EVT clusters (Clusters 8 and 1) were defined by a gene set of EVT‐specific markers (FN1, ITGA1, MMP9, MMP2, and CCL2) (Figure 3e). We defined Clusters 10 and 11 as STBs based on the lineage‐specific markers ERVW‐1, CGA, SDC1, S100P, GCM1, SLC13A4, CYP19A, and GDF15 (Figure 3f).

Next, unsupervised lineage trajectory analysis were performed to reconstruct the lineage relationships using monocle 2 (Figure S3a–d). The time points of the differentiation process match well with the pseudotimes (Figure S3b). We then examined the top 50 differentially expressed genes (DEGs) in the bifurcation of the first branchpoint. The results indicated that these genes enriched in this branchpoint were tightly related with placental trophoblast development, such as KRT8, KRT18, and CD9 (Figure S3d). It revealed that the trophoblast lineages within the 3D cultures seemed to share common origins during the process of placenta development.

In addition, the placenta‐like organoids at 9 and 24 days of differentiation were also individually analyzed as shown in Figures S4 and S5. Based on the specific markers of different cell types, D9 sample clusters were annotated and refined into five broad classes of cells: CTBs, STBs, EVTs, proliferating cells, and vascular cells (Figure S4a). The UMAP and violin plots demonstrate the expression atlas of representative marker genes (Figure S4b–g). The D24 samples were also clustered into five broad clusters: CTBs, STBs‐like cells, EVTs, proliferating cells, and vascular cells, according to the established markers (Figure S5a–f). However, the STBs cell seem to be a minority. Altogether, the single‐cell survey indicated this hiPSC‐derived 3D tissue contained the basic cell types of human placenta: trophoblasts and endogenous vascular cells. This result reflects the possibility of differentiating hiPSCs into vascular‐like tissues in a 3D culture system, which has not been previously achieved in 2D cultures or stem cell‐based in vitro models.

### 2.3 Morphological characterization of hiPSC‐derived placenta‐like tissue

**FIGURE 4:**
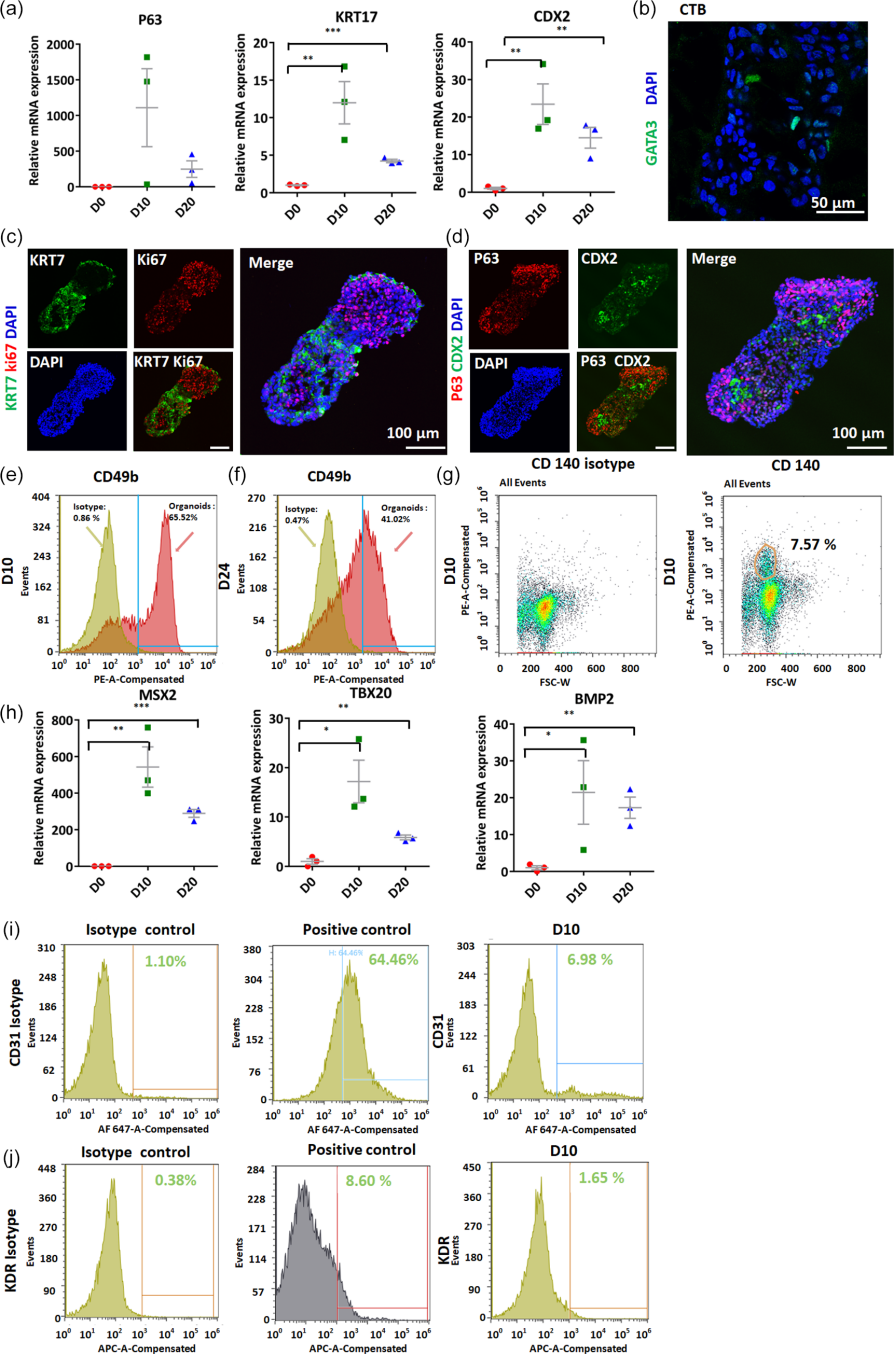
Expression of CTB‐, endothelial cell‐, and pericyte‐related markers in the formed placenta‐like 3D tissue. (a) qRT‐PCR demonstrating the relative mRNA expression of CDX2, P63, and KRT17 in the 3D tissue at 0, 10, and 20 days of differentiation (*n* = 3). Student's *t*‐test were used for data analysis (*p < 0.05; **p < 0.01; ***p < 0.001). (b) GATA3 staining of the 3D tissue at Day 16, which revealed the expression of a trophoblast marker in the 3D culture. (c and d) Immunofluorescence analysis for the trophoblast marker KRT7, proliferative cell marker Ki67, and trophoblast stem cell markers CDX2 and P63 in Day 16 3D tissue. (e–g) Flow cytometry analysis of Days 10 and 24 3D tissue stained for CD140 and CD49b to identify proliferating trophoblast progenitor cells and pericytes, respectively. (h) qRT‐PCR to identify mesodermal markers at 0, 10, and 20 days of differentiation. Three independent experiments were conducted. The data were analyzed using Student's *t*‐test (*p < 0.05; **p < 0.0; ***p < 0.001). (i and j) Flow cytometry analysis of the endothelial cell marker CD31 (i) and the vascular progenitor marker KDR (j) in the tissue at 10 days. Primary HUVECs served as a positive control.

To further examine the cellular components and tissue structure in detail, we performed a histological analysis of the placenta‐like organoids. The mRNA expression of trophoblast and mesoderm‐associated genes in the 3D tissue was probed by quantitative real‐time reverse transcription polymerase chain reaction (qRT‐PCR) (Figure 4a,h). The results showed that trophoblast and mesoderm markers were significantly upregulated, reflecting the coordinated induction of trophoblast and mesodermal lineages in the 3D culture system. Moreover, the confocal microscopy images revealed CTB differentiation in the 3D tissue, which was characterized by the expression of the CTB‐associated proteins KRT7, Ki67, GATA3, P63, and CDX2 (Figure 4b–d). Flow cytometry analysis was conducted to determine the percentage of ITGA2^+^ cells in the 3D cultures at different days of differentiation. In the human placenta, ITGA2 represent a trophoblast progenitor niche at the base of CTB cell columns.^34^ The majority cell type within the 3D tissue was ITGA2^+^ cells (Figure 4e). The percentage of trophoblast progenitor niche gradually decreased because the placenta‐like tissue differentiated into other cell types (Figure 4f). Vascular cells, including PDGFRβ^+^ pericytes and CD31^+^ endothelial cells, were also identified in the 3D tissue (Figure 4g,i). Notably, the percentage of placental PDGFRβ^+^ pericytes dramatically decreased at Day 24 because SB431542 inhibited pericyte differentiation (Figure 4g). A previous study indicated that FLT1 and kinase insert domain receptor (KDR) are vascular progenitors and have individual contributions to human fetoplacental endothelial cell‐mediated angiogenesis in uncomplicated pregnancies.^35^ We next detected the expression of KDR in the 3D tissue at Days 8 and 12. It appeared that a small population of cells expressed the vascular progenitor marker KDR (Figure 4j). With the emergence of KDR^+^ progenitors and upregulated expression of CD31^+^ endothelial cells, the 3D tissue exhibited vascular specification seen in self‐organized placenta‐like organoids.

**FIGURE 5:**
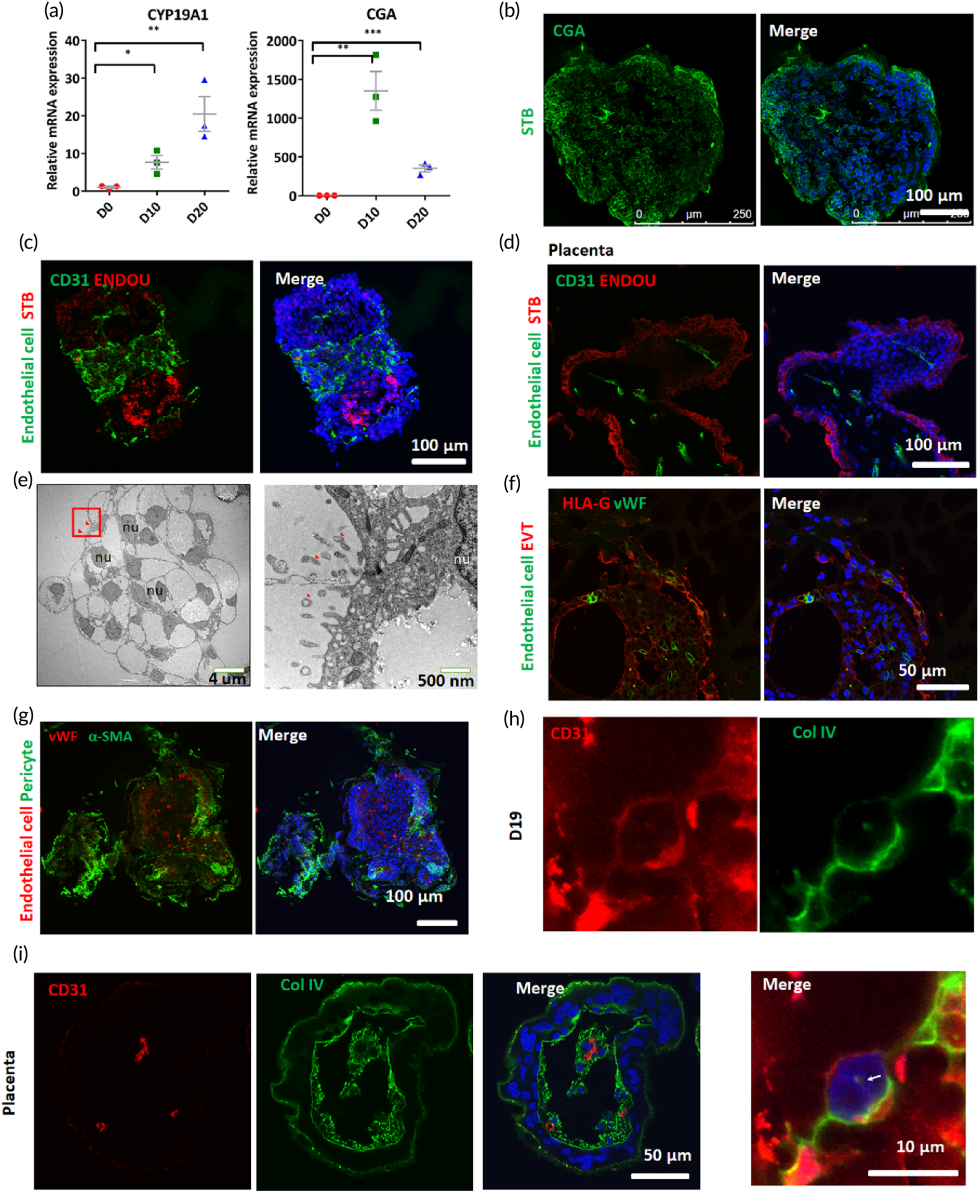
Characterization of STB and EVT in the placenta‐like tissue. (a) The relative mRNA expression of STB markers CYP19A1 and CGA in Day 0, 10, and 20 placenta‐like organoids. Data were analyzed using Student's *t*‐test (*p < 0.05; **p < 0.01). (b and c) Confocal micrographs showing the expression of the endothelial cell marker CD31 and STB markers ENDOU and CGA in the 3D tissue at 16–20 days. (d) Immunofluorescence images showing CD31 and ENDOU expression in primary placenta (*n* = 3; 6–8 weeks gestation in first‐trimester). (e) Electron transmission microscopy images of multinucleated STBs. The right picture showed the enlarged square areas in the left picture. The microvilli were indicated by red arrowheads. nu, nucleus. (f) Expression of the endothelial cell marker vWF and the EVT marker HLA‐G in day 19 3D cultures detected by immunofluorescence analysis. (g) Staining of Day 19 3D tissue for vWF and αSMA, markers of endothelial cells and pericytes, respectively. (h and i) CD31 staining to detect small endothelial tubes in Day 19 tissue (h) and primary first‐trimester tissue (i). The endothelial cells appeared to be covered by extracellular matrix collagen type IV (Col IV). Scale bars are indicated in the images.

The above culture conditions appear to have contributed to the differentiation of CTBs. To determine whether these conditions could provide an instructive signal for CTB differentiation into EVTs and STBs, we investigated the relative expression level of STB‐ and EVT‐associated genes in the 3D tissue at different days using qRT‐PCR and immunofluorescence staining. Transcript analysis of the STB‐specific genes CGA, and CGB was conducted using qRT‐PCR. We observed the upregulated expression of STB‐related genes, suggesting the appearance of STB^+^ populations within the formed 3D cultures (Figure 5a). Moreover, the immunofluorescence staining revealed the differentiation of STBs with the specific markers CGA and ENDOU (Figure 5b–c). The expression of the STB‐specific marker ENDOU was consistent with observations of the primary placenta‐like organoids (6–8 weeks gestation) (Figure 5d). During placentation in vivo, STBs form as multinucleated cells that constitute the brush border between the maternal and fetal circulations. Here, cells with surface microvilli, the characteristic features of trophoblast cells, were detected by transmission electron microscopy (TEM) (Figure 5e).

In addition to STBs, EVTs are a key trophoblast subtype that invades into the maternal decidua to transform the spiral arteries.^1^ The classical feature of EVT differentiation is the upregulated expression of the nonclassical MHC Class I molecule HLA‐G.^36^ To identify the EVT lineage, we detected HLA‐G^+^ EVTs in 3D cultures via immunofluorescence staining, revealing the differentiated EVT lineage within the placenta‐like organoids (Figure 5f). Pericyte cell marker: SMA was observed in Day 19 3D tissue (Figure 5g). In particular, the confocal imaging indicated the formation of complex and interconnected vessel‐like networks with CD31^+^ endothelial tubes (Figure 5h), which was in line with the primary tissue (Figure 5i).

### 2.4 Functional characterization of placenta‐like tissue derived from hiPSCs

**FIGURE 6:**
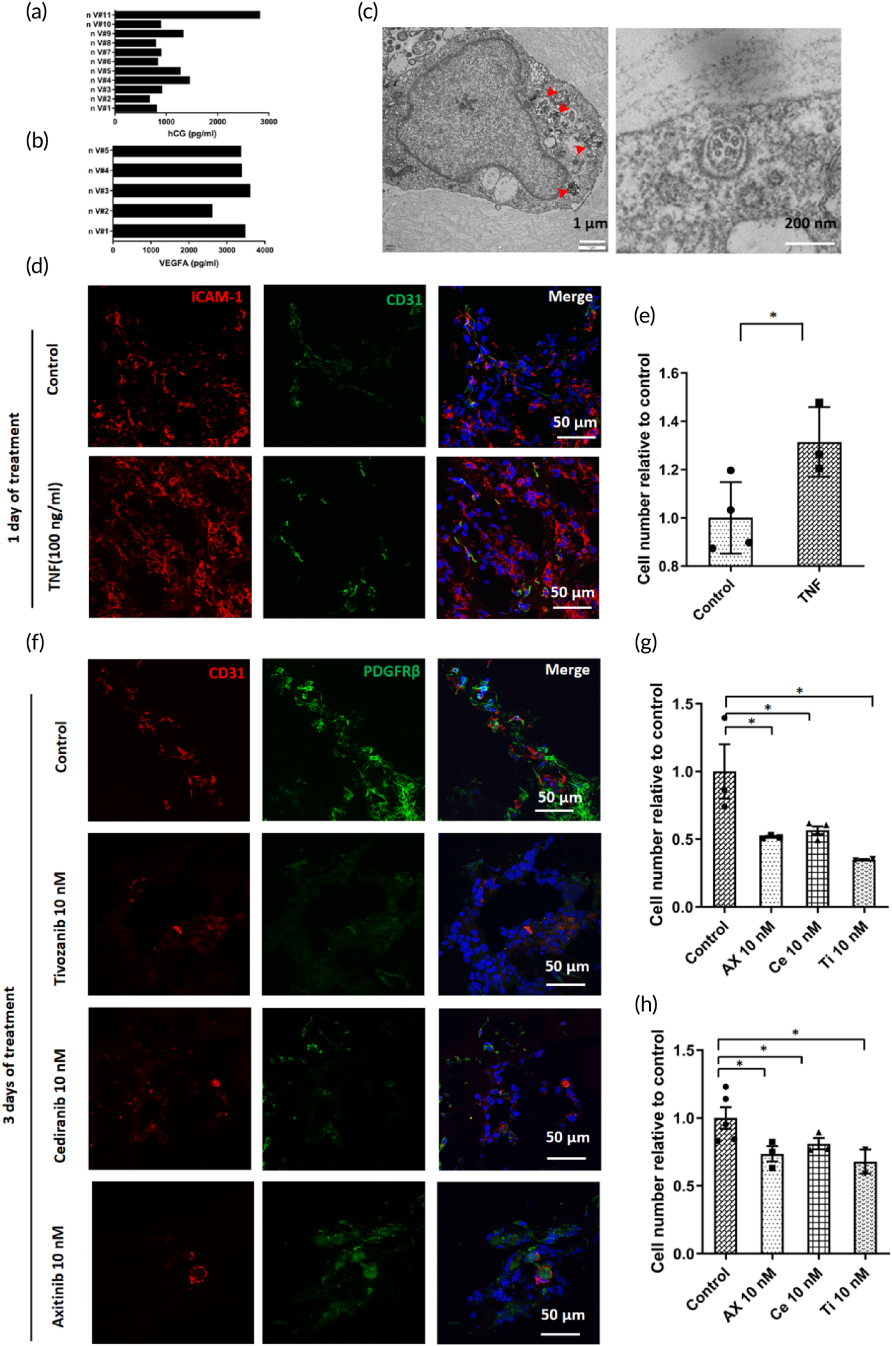
Functional characterization of placenta‐like tissue containing endogenous vascular cells. (a) Secretion of hCG‐β by the 3D tissue (*n* = 11). (b) Secretion of VEGFA from the formed 3D tissues (*n* = 5). (c) Electron transmission microscopy images of endothelial cells. Red arrowheads indicate Weibel–Palade bodies in the endothelial cells. (d) TNF‐α‐mediated activation of placental tissues detected by the induction of ICAM‐1 expression in endothelial cells (CD31). ICAM‐1 expression was determined after 24 h of TNF‐α treatment (100 ng/ml). Experiments were independently repeated three times with similar results. DAPI was used to counterstain the nuclei. (e) Quantitative analysis of the ICAM‐1 fluorescence intensity in the formed tissue after exposure to TNF‐α stimulation. Data are presented as the mean ± SEM. The data were analyzed using Student's *t*‐test (*p < 0.05). (f) Total immunofluorescence analysis of the placenta‐like tissue (Day 17) treated with VEGFR inhibitors for 3 days. Scale bar = 50 μm. (g and h) Quantitative analysis of PDGFβ and CD31 fluorescence intensity in the 3D tissue under different conditions. Data are presented as the mean ± SEM. The data were analyzed using Student's *t*‐test (*p < 0.05)

The in vivo placenta secretes a series of hormones and growth factors to maintain placental development and fetal growth during pregnancy. Human chorionic gonadotropin‐β (hCG‐β) is a hormone that is mainly produced by STBs and serves as a critical marker for pregnancy tests.^37^, ^38^ Vascular endothelial growth factor A (VEGFA) is an important signal protein in regulating the early formation of blood vessels in human placenta.^39^We identified hCG‐β and VEGFA in the medium of the 3D cultures by enzyme‐linked immunosorbent assay, indicating the secretory ability of the produced placenta‐like organoids in vitro (Figures 6a,b).

A unique feature of this 3D model system is the presence of endogenous vascular cells that are not found in 2D cultures. TEM and immunofluorescence staining were conducted to further characterize the function of the vascular‐like network. The TEM analysis demonstrated the existence of Weibel–Palade bodies in the cytoplasm, which are a key feature of endothelial cells within the placenta‐like organoids (Figure 6c).

To determine the response of the placenta‐like organoids to inflammatory factors, the 3D cultures were exposed to tumor necrosis factor‐α (TNF‐α). Increased expression of the adhesion protein ICAM‐1 was observed after TNF‐α stimulation (Figure 6d,e), reflecting the feasibility of this placental model to recapitulate the human‐relevant responses to an inflammatory stimulus. After treatment with VEGF receptor inhibitors, which are known to disrupt VEGFA signaling, the vascular‐like network was compromised as indicated by decreased CD31 and PDGFβ expression (Figure 6f,h). These results demonstrated the mature function of the vascular cells within the 3D tissue, which could respond to external stimuli.

## 3 DISCUSSION AND PERSPECTIVE

In this study, we established a defined protocol to engineer hiPSC‐derived placenta‐like tissue containing endogenous vascular cells in a defined 3D culture system. The generated placental organoids were able to recapitulate the multicellular components and functional features of first‐trimester human placental tissue. By exposing the culture to a cohort of chemical factors, the hiPSCs could self‐organize into 3D tissues with trophoblast lineages (CTBs, STBs, and EVTs) and vascular lineages in the 3D culture, as identified by scRNA‐seq, qRT‐PCR, immunofluorescence, and flow cytometry analysis. In particular, we identified endogenous vascular cells, such as endothelial cells and pericytes, that have not been observed in 2D cultures. Moreover, the 3D tissue could secrete both the placental‐specific hormone hCG‐β and blood vessel‐related VEGFA. These capabilities reflect the physiologically relevant functional aspects of this human placenta‐like organoid.

In vivo, a complex vascular network is required to ensure sufficient oxygen and nutrient transfer from the placenta to the fetus during pregnancy. In this study, we established a 3D human placental model containing both a mesoderm lineage related to placental vasculature and a functional trophoblast lineage. The 3D cultures were characterized by the inclusion of endogenous vascular cells, initiate angiogenesis, and promote endothelial cell formation of tube‐like structures. Upon stimulation with inflammatory factors and a VEGFA inhibitor, the vascularized‐like structure exhibited upregulated expression of the adhesion protein ICAM‐1 and compromised tube‐like structures, indicating the mature function of the vascular‐like cells in the 3D tissue. Placental pericytes are reportedly associated with vascular stabilization. We ascertained the presence of pericyte differentiation in our culture system from D9 to D19. However, the large number of pericytes might inhibit the construction of vessel structures. This finding is in line with a previous study in which placental pericytes inhibited vessel growth in terminal villous microvessels.^40^

Trophoblast organoids derived from first‐trimester placenta and hPSCs provide a near‐physiological model because they resemble the villous placenta in vivo. This model has enabled the study of maternal–fetal interactions that occur during human placentation.^21^, ^22^, ^23^, ^24^, ^41^ However, the human placenta is a complex vascularized organ that contains the trophoblast as well as other placental components, such as vascular cells. In this study, we made the first attempt to create a human placental model from hiPSCs with a vascular‐like network that included both the trophoblast and vascular lineages. This 3D model system has the potential to be used to study interactions between trophoblasts and other placental cells and to explore the maternal–fetal interface of early pregnancy. It might also be used to probe gestational diseases associated with impaired vascular networks, such as fetal growth restriction and pre‐eclampsia. In addition, it can serve as a potential platform for drug testing and for studying pathogen infection during pregnancy.

Considering that Matrigel hydrogel matrix were used to generate organoids such as brain, kidney, and liver, we establish placenta‐like organoids in this study using Matrigel. Due to Matrigel suffering from high batch‐to‐batch variability, we will aim to develop a more controlled hydrogel matrix to replace Matrigel in future work. Moreover, it is essential to see if the trophoblast organoids and cells function differently without signaling from the vascular cells. It is reasonable to introduce exogenous endothelial cells to probe whether the function of trophoblast organoids is different in the presence or absence of vascular cells. Owing to the complex structure and function of the human placenta, further work is still needed to build more advanced model systems by integrating bioengineering strategies.^42^ Various engineering approaches such as microfluidics and biomaterials could have added value by providing finely tuned control of mechanical and biochemical cues within the cellular microenvironment for the generation of perfused vasculature. The incorporation of mechanical and biochemical cues, such as extracellular matrix stiffness and dynamic flow in the 3D culture system, could contribute to the creation of higher fidelity placenta models, thus facilitating the understanding of human placenta biology in healthy and compromised pregnancy.

As placental stromal cells such as pericytes and fibroblasts play critical roles in maintaining the long‐term growth of in vitro 3D microvessels,^40^ fibroblasts should be added to 3D cultures to engineer more stable vessel structures. In addition, the maternal signaling is critical for to establish a truly functional system for the maternal–fetal interface. This work can be extended to probe the interactions between the trophoblast organoids and decidual NK cells by cell coculture in a microfluidic system in our subsequent study. We envision that these synthetic strategies will open new avenues to create high‐fidelity human placental 3D model systems that can contribute to the study of placental biology and disease etiology and could be used in safety testing and have translational applications.

## 4 EXPERIMENTAL SECTION

### 4.1 Human tissue

Ethical approval was granted by the Ethics Committee of Dalian Municipal Women and Children's Medical Center (2019005). Based on the guiding principles in the Declaration of Helsinki of the World Medical Association, written informed consent was obtained from all participants. All primary placenta samples (6–8 weeks of gestation in first trimester) utilized here were taken from patients who chooses the selective termination of normal pregnancy (vacuum suction). The obtained placenta tissue samples were treated with NaCl (0.9%) and immediately stored in phosphate‐buffered saline (PBS).

### 4.2 hiPSCs culture

hiPSCs generated from reprogramed skin fibroblasts were maintained under feeder‐free conditions in accordance with our previous study.^29^ Two hiPSC lines were cultured on Matrigel‐coated plates in mTeSR1 medium and passaged at the ratio of 1:6 to 1:5 with Accutase every 4–5 days.

### 4.3 Formation of hiPSC‐derived placenta‐like endogenous vascular cells

To generate placenta‐like organoids, hiPSCs were digested into small pellets (containing several cells) using Accutase and resuspended in Knockout Serum Replacement (KSR) medium supplemented with DMEM: F12 medium, 20% KOSR (Knockout Serum Replacement), 1X GlutaMax, 1X nonessential amino acid (NEAA), and 1X penicillin–streptomycin. Next, 5 × 10^6^ cells were seeded onto a micropillar chip for cell aggregation as previously described^43^ and maintained in 5% O_2_. On Day 2, the cell aggregates were treated with 10 μM BMP4 in placental induction (DMEM: F12 medium, 100 μg/ml Primocin, 1X penicillin–streptomycin, 1X GlutaMax, 1.25 mM *N*‐acetyl‐l‐cysteine, 1X B27 minus VA, 1X N_2_, and 1X NEAA). On Day 4, the aggregates were further treated with 10 μM BMP4 in placental differentiation medium (DMEM: F12 medium), 100 ng/ml FGF2 (human FGF basic), 50 ng/ml human hepatocyte growth factor (HGF), 80 ng/ml R‐spondin 1, 2.5 μM Prostaglandin E2, 500 nM A83‐01, 1.5 μM CHIR99021, 50 ng/ml EGF, 25 μM Y‐27632, 100 ng/ml VEGFA (Table S1). From Days 6 to 20, 10 μM SB43152 was added to suppress pericyte differentiation. On Day 12, the cell aggregates were embedded into Matrigel and cultured in a 21% O_2_ environment. From Day 20, the cells were cultured in placental maturation medium (PMM: DMEM: F12 medium, 15% FBS, 1X GlutaMax, 1X NEAA, 1X penicillin–streptomycin with 50 ng/ml FGF2, 50 ng/ml VEGFA, and 5 μM Y‐27632). All media were changed every 2–3 days. The 3D cultures were either directly analyzed or extracted from the gels for subsequent analysis. The placenta‐like tissue could be cultured for more than 1 month.

### 4.4 Cryopreservation ang immunofluorescence staining

The tissue cryopreservation and immunofluorescence staining procedures are described in detail in previous study.^43^ In brief, 3D cultures harvested at different days were fixed with 4% paraformaldehyde for approximately 30 min at room temperature. They were then washed three times with PBS and transferred to 30% sucrose overnight for dehydration. On Day 2, the placenta‐like tissues were embedded in optimal cutting temperature compound (Sakura) and then cryosectioned into 15–20 μm sections using a Leica CM1950 cryostat. Before conducting the immunofluorescence staining, we washed the cryosectioned slices with PBS and then permeabilized the frozen sections with Triton X‐100 (0.2%), followed by blocking with 10% goat serum (ZSGB‐Bio) for 1 h at 37°C. After blocking, the sections were washed with PBS and then incubated with primary antibodies overnight at 4°C, followed by staining with secondary antibodies for 1 h at 37°C. The antibodies used are described in detail in Table S2. The sample images were captured using a confocal microscope (Olympus) after counterstaining the nuclei with DAPI.

### 4.5 Enzyme‐linked immunosorbent assay

The levels of VEGFA and hCG‐β secreted by the established placenta‐like tissue were detected using human VEGFA (DY293B; R&D) and hCGβ ELISA kits (ab100533; Abcam) according to the manufacturer's instructions. After culturing for 48 h, the collected culture medium was centrifuged to remove debris. Then the supernatant was stored at −80°C before use. The absorbances were measured using TECAN Infinite M Nano.

### 4.6 Flow cytometry

The Matrigel‐embedded clusters were dissociated from the matrix using Cell Recovery Solution (354253; Corning). Organoids were dissociated by incubating in trypsin (0.25%) at 37°C for about 10 min, followed by incubation at 4°C for 20 min to depolymerize the Matrigel. Following incubation, the cells were washed with medium containing FBS, and then 40‐μm cell strainers (2340; Falcon) were used to obtain single cells. The cells and isotype‐matched controls were stained with ITGA2–PE, KDR, CD140 (PDGFRβ), and CD31 for analysis (Table S2). The viability of each cell type was measured by Live/Dead Fixable Near‐IR Dead Cell Stain (L10119; Life Technologies). FACS analysis were performed by SH800S cell sorter (Sony Biotechnology).

### 4.7 Transmission electron microscopy

Collected samples were fixed with 2.5% polyformaldehyde and glutaraldehyde in PBS (pH 7.4) for 1 h at room temperature. The 3D cultures were post‐fixed in 1% osmium tetroxide (0.1 mol/L sodium cacodylate) for 1.5 h. They were stained with 1% (wt/vol) uranyl acetate in double‐distilled water for 50 min. The samples were thoroughly washed and dehydrated. They were embedded in Embed 812 resin and polymerized for 48 h at 60°C. The polymerized samples were trimmed by Leica ultramicrotome EM UC6 (Leica) and then sliced to 50‐nm sections. The sliced samples were collected on EM grids that were coated with carbon film. Images of the TEM sample grids were captured with a Spirit transmission electron microscope (FEI) operated at 100 kV.

### 4.8 RNA extraction and real‐time reverse transcription PCR

The RNA extraction and PCR procedures are described in detail in previous study.^43^ In brief, total mRNA from the 3D tissue or hiPSCs were extracted using Trizol reagent. The RNA concentration and quality were measured using NanoPhotometer (Implen). cDNA was generated and then amplified using Ex Taq DNA polymerase (Takara). The amplification conditions were listed as following: 1 min of denaturing at 94°C, 45 s of annealing at 58°C, and 30 s of extension at 72°C, with 40 cycles. Table S3 lists the primer pairs that were used. Real‐time PCR was conducted on a PikoReal 96 real‐time PCR system (Thermo Fisher Scientific).

### 4.9 scRNA‐seq data processing

Seurat (version 3.0) was used to conduct quality control and filtering. The quality control parameters to retain cells were defined as a mitochondrial gene content of less than 10%. The quality control and filtering results are shown in violin plots (Figure S6). After rigorous quality control and filtering, 6744 single cells from Days 9 and 10, 259 single cells from Day 24 remained. Unsupervised lineage trajectory analysis on a down‐sampled set of Days 9 and 24 cells was then performed using Monocle 2 (version 2.10.0). Briefly, the genes used to order cells along the trajectory were the genes whose mean expression *R* value was 0.05. The branched expression analysis modeling function of Monocle 2 was used to identify DEGs (significantly DEGs) (*q* value % 1e−4) at branch points along the trajectory. In addition, a branched heatmap was used to visualize significant branch‐specific DEGs to detect changes in both cell fates concurrently. Hierarchical clustering of branch‐specific DEGs was used to identify genes with similar lineage‐dependent expression patterns, which were then classified into upregulated or downregulated branch‐specific DEGs.

### 4.10 Statistics and reproducibility

All experiments described in this work were repeated, and similar results were obtained from independent samples. Data are presented as the mean ± SEM. For statistical significance, *p < 0.05 was accepted as statistically significant. At least 15 formed tissue samples in each group were randomly investigated by qRT‐PCR, immunohistochemical staining, and quantitative analysis.

## AUTHOR CONTRIBUTIONS

**Kangli Cui:** Conceptualization (equal); formal analysis (equal); investigation (equal); methodology (equal); project administration (equal); software (lead); writing – original draft (lead); writing – review and editing (lead). **Tingwei Chen:** Methodology (equal); software (equal); writing – review and editing (equal). **Yujuan Zhu:** Conceptualization (equal); data curation (equal); investigation (equal); project administration (equal); software (equal); writing – original draft (equal); writing – review and editing (equal). **Yang Shi:** Resources (supporting). **Yaqiong Guo:** Visualization (supporting). **Jianhua Qin:** Conceptualization (lead); data curation (lead); formal analysis (lead); funding acquisition (lead); investigation (lead); methodology (lead); project administration (lead); resources (lead); supervision (lead); validation (lead); visualization (lead); writing – original draft (lead); writing – review and editing (lead).

## CONFLICT OF INTERESTS

The authors declare no competing interests.

### PEER REVIEW

The peer review history for this article is available at https://publons.com/publon/10.1002/btm2.10390.

## Supporting information


**Appendix S1** Supporting information
**Figure S1**. Viability characterization of 3D cultures with or without Matrigel at day 20. a, Bright‐field images showing protrusions from the placenta‐like tissue within the Matrigel. b, Flow cytometry analysis of dead cells in placenta‐like tissue cultured with (w) or without (w/o) Matrigel after 20 days of differentiation. The results showed that the 3D tissue grew well in the Matrigel.
**Figure S2**. Optimized protocols to generate placenta‐like tissue in different culture conditions. Vascular‐like networks and cell components and proportions were examined by fluorescence immunostaining and flow cytometry analysis. ↑, increase; ↓, decrease; O, no significant change. VEGFA and TGF‐β inhibitor SB431542 were used to promote the generation of endothelial cells. The proportions of cell types in the 3D clusters were further analyzed after the addition of SB431542. 3D clusters with pericyte differentiation appeared to be inhibited by SB431542 from day 6 to 20. As these chemical factors may contribute to trophoblast differentiation and proliferation, we examined the derivation of cell lineages by individually changing the major components of the culture medium. The culture conditions were optimized to differentiate the 3D clusters by adjusting chemical factors in the condition (R‐spondin 1, HGF, bFGF, and EGF). The results showed that reduced/fewer chemical factors led to an increased number of pericytes and fewer CTBs and endothelial cells, revealing the critical role of chemical factors in adjusting the percentages of the different cell types in the 3D culture.
**Figure S3**. Single‐cell transcriptome atlas of the 3D tissue formed from hiPSCs. a, Lineage trajectory of cell sub‐cluster by sample. b‐c, Lineage trajectory of cell sub‐cluster by pseudotime and cell state. d, Heatmap of branched expression of the top 50 DEGs at branch point 1, as indicated in panel d.
**Figure S4**. Single‐cell expression altas of placenta‐like tissue at D9. a, UMAP plot showing 6507 cells from D9 tissue. Unsupervised clustering identified 11 clusters labeled by diverse colors. The 15 clusters were classified into six cell types based on the expression of cell‐specific markers. b‐g, Violin plot demonstrating different markers related to CTBs, vascular cells, pluripotent cells, EVTs, and proliferating cells.
**Figure S5**. Single‐cell expression atlas of placenta‐like tissue at D24. a, UMAP plot demonstrating 9804 cells from D24 placenta‐like organoids. Unsupervised clustering identified 13 clusters marked by diverse colors. The 13 clusters were classified into five cell types based on the expression of cell‐specific markers. b‐f, Violin plots displaying the expression of specific genes among the different cell types in the placenta‐like tissue: proliferating cells, CTBs, STBs, EVTs, vascular cells, and STBs.
**Figure S6**. Quality control and filtering of the 3D culture single‐cell transcriptome atlas at day 9 and day 24.
**Table S1**. List of chemical factors used to generate placenta‐like tissues.
**Table S2**. List of primary antibodies used in immunofluorescence staining (IF) and flow cytometry (FC).
**Table S3**. Primers pairs used to detect the mRNA expression.

## Data Availability

The raw scRNA‐seq data files of this work have been deposited and available in the National Center for Biotechnology Information Sequence Read Archive repository (https://dataview.ncbi.nlm.nih.gov/object/PRJNA694235?reviewer=dvcl9ohi7i8g0ehh3amh9ef001) with accession number PRJNA694235. Other data supporting the results of this work can be obtained from the corresponding author.
