# Supplementary material for: Engineering placenta‐like organoids containing endogenous vascular cells from human‐induced pluripotent stem cells
Source: Bioeng Transl Med. 2022 Sep 23;8(1):e10390. doi: 10.1002/btm2.10390 (PMC9842056; doi:10.1002/btm2.10390)
Supplement: Supplementary file 1 — Appendix S1 Supporting information Figure S1. Viability characterization of 3D cultures with or without Matrigel at day 20. a, Bright‐field images showing protrusions from the placenta‐like tissue within the Matrigel. b, Flow cytometry analysis of dead cells in placenta‐like tissue cultured with (w) or without (w/o) Matrigel after 20 days of differentiation. The results showed that the 3D tissue grew well in the Matrigel. Figure S2. Optimized protocols to generate placenta‐like tissue in different culture conditions. Vascular‐like networks and cell components and proportions were examined by fluorescence immunostaining and flow cytometry analysis. ↑, increase; ↓, decrease; O, no significant change. VEGFA and TGF‐β inhibitor SB431542 were used to promote the generation of endothelial cells. The proportions of cell types in the 3D clusters were further analyzed after the addition of SB431542. 3D clusters with pericyte differentiation appeared to be inhibited by SB431542 from day 6 to 20. As these chemical factors may contribute to trophoblast differentiation and proliferation, we examined the derivation of cell lineages by individually changing the major components of the culture medium. The culture conditions were optimized to differentiate the 3D clusters by adjusting chemical factors in the condition (R‐spondin 1, HGF, bFGF, and EGF). The results showed that reduced/fewer chemical factors led to an increased number of pericytes and fewer CTBs and endothelial cells, revealing the critical role of chemical factors in adjusting the percentages of the different cell types in the 3D culture. Figure S3. Single‐cell transcriptome atlas of the 3D tissue formed from hiPSCs. a, Lineage trajectory of cell sub‐cluster by sample. b‐c, Lineage trajectory of cell sub‐cluster by pseudotime and cell state. d, Heatmap of branched expression of the top 50 DEGs at branch point 1, as indicated in panel d. Figure S4. Single‐cell expression altas of placenta‐like tissue at D9. a, UMAP pl [file BTM2-8-e10390-s001.docx]

**Supporting information**


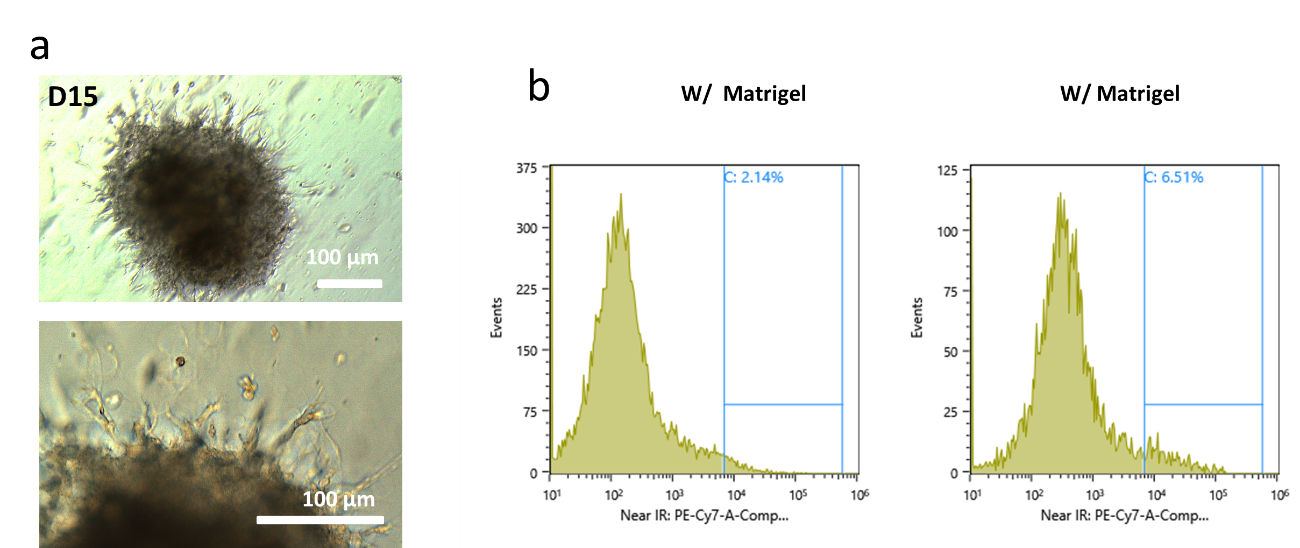


**Figure S1. Viability characterization of 3D cultures with or without Matrigel at day 20.** **a**, Bright-field images showing protrusions from the placenta-like tissue within the Matrigel. **b**, Flow cytometry analysis of dead cells in placenta-like tissue cultured with (w) or without (w/o) Matrigel after 20 days of differentiation. The results showed that the 3D tissue grew well in the Matrigel.


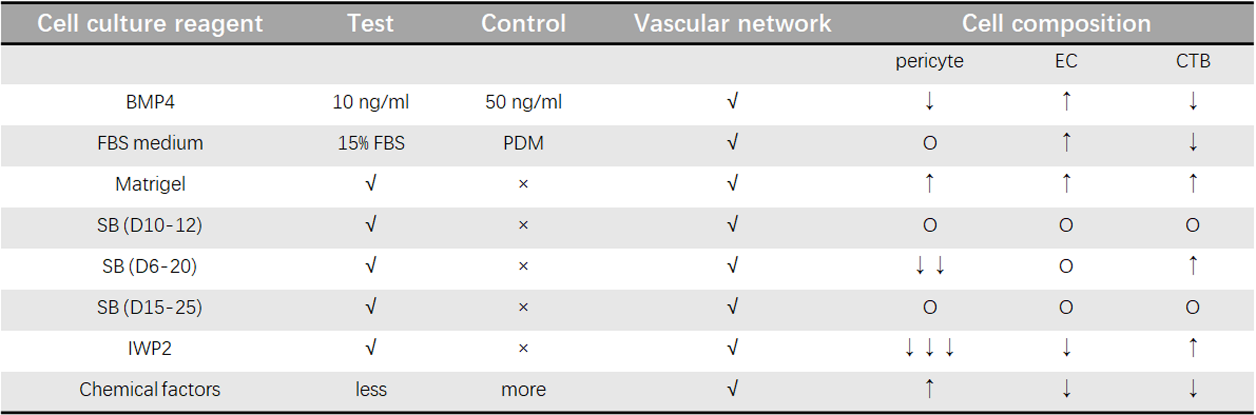


**Figure S2. Optimized protocols to generate placenta-like tissue in different culture conditions.** Vascular-like networks and cell components and proportions were examined by fluorescence immunostaining and flow cytometry analysis. ↑, increase; ↓, decrease; O, no significant change. VEGFA and TGF-β inhibitor SB431542 were used to promote the generation of endothelial cells. The proportions of cell types in the 3D clusters were further analyzed after the addition of SB431542. 3D clusters with pericyte differentiation appeared to be inhibited by SB431542 from day 6 to 20. As these chemical factors may contribute to trophoblast differentiation and proliferation, we examined the derivation of cell lineages by individually changing the major components of the culture medium. The culture conditions were optimized to differentiate the 3D clusters by adjusting chemical factors in the condition (R-spondin 1, HGF, bFGF, and EGF). The results showed that reduced/fewer chemical factors led to an increased number of pericytes and fewer CTBs and endothelial cells, revealing the critical role of chemical factors in adjusting the percentages of the different cell types in the 3D culture.


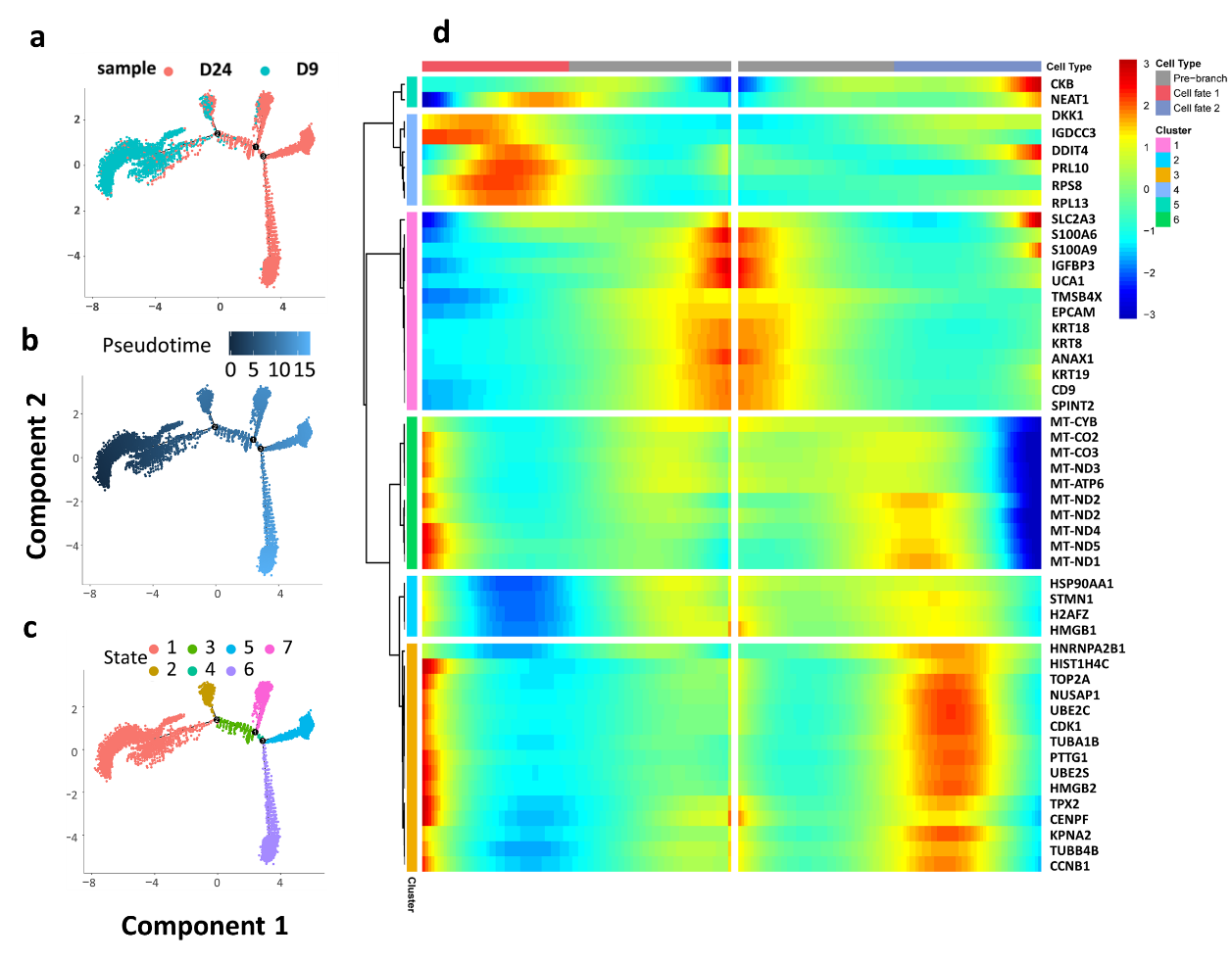


**Figure S3. Single-cell transcriptome atlas of the 3D tissue formed from hiPSCs. a**, Lineage trajectory of cell sub-cluster by sample. **b-c***,* Lineage trajectory of cell sub-cluster by pseudotime and cell state. **d**, Heatmap of branched expression of the top 50 DEGs at branch point 1, as indicated in panel **d**.

**
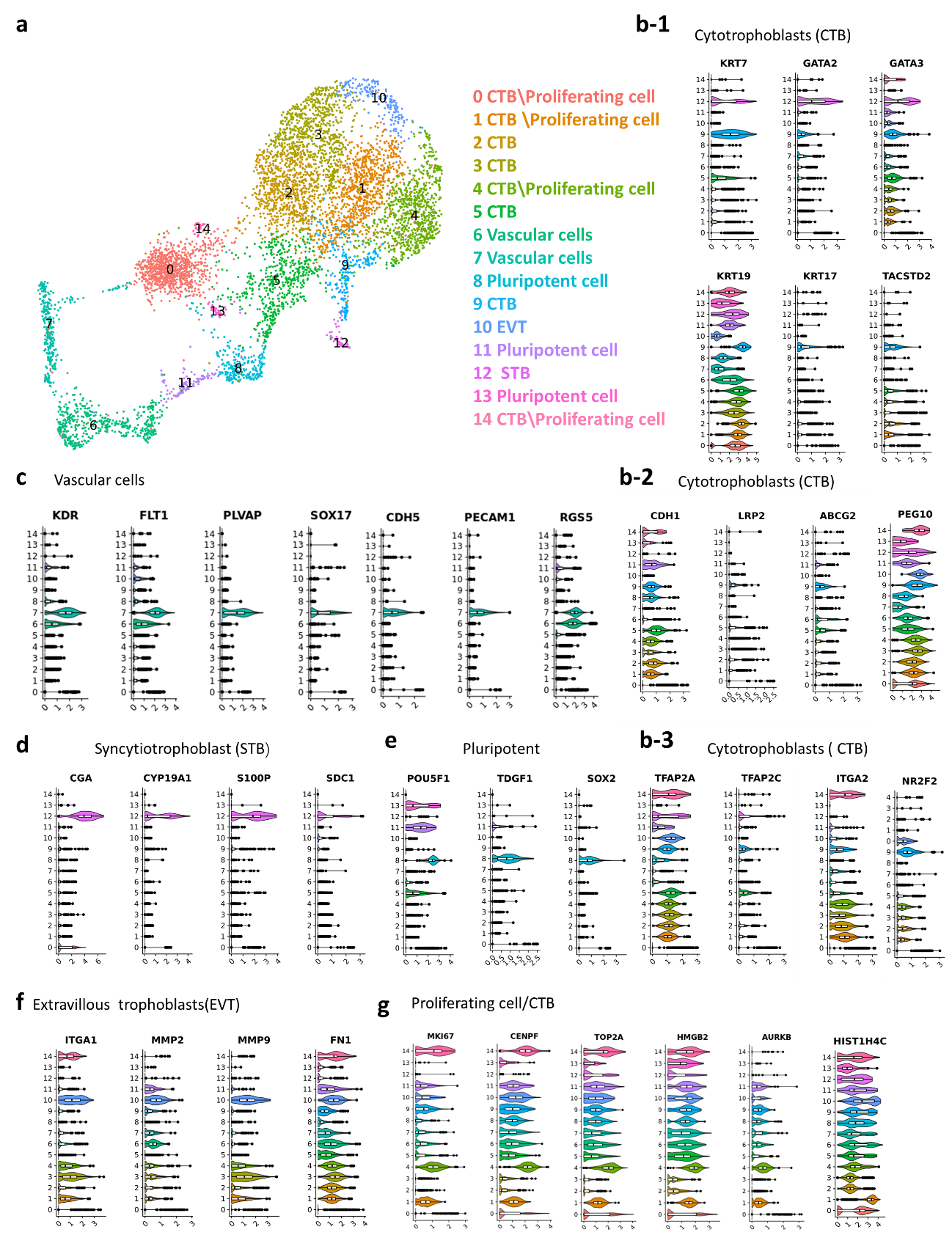
**

**Figure S4. Single-cell transcriptomics analysis of placenta-like tissue at D9. a**, UMAP plot displaying 6507 cells from tissue on D9 of differentiation. Unsupervised clustering identified 11 clusters, which are marked by different colors. The 15 clusters were classified into six cell types based on the expression of cell-specific markers. **b-g**, Violin plot showing the markers related to CTBs, vascular cells, pluripotent cells, EVTs, and proliferating cells.


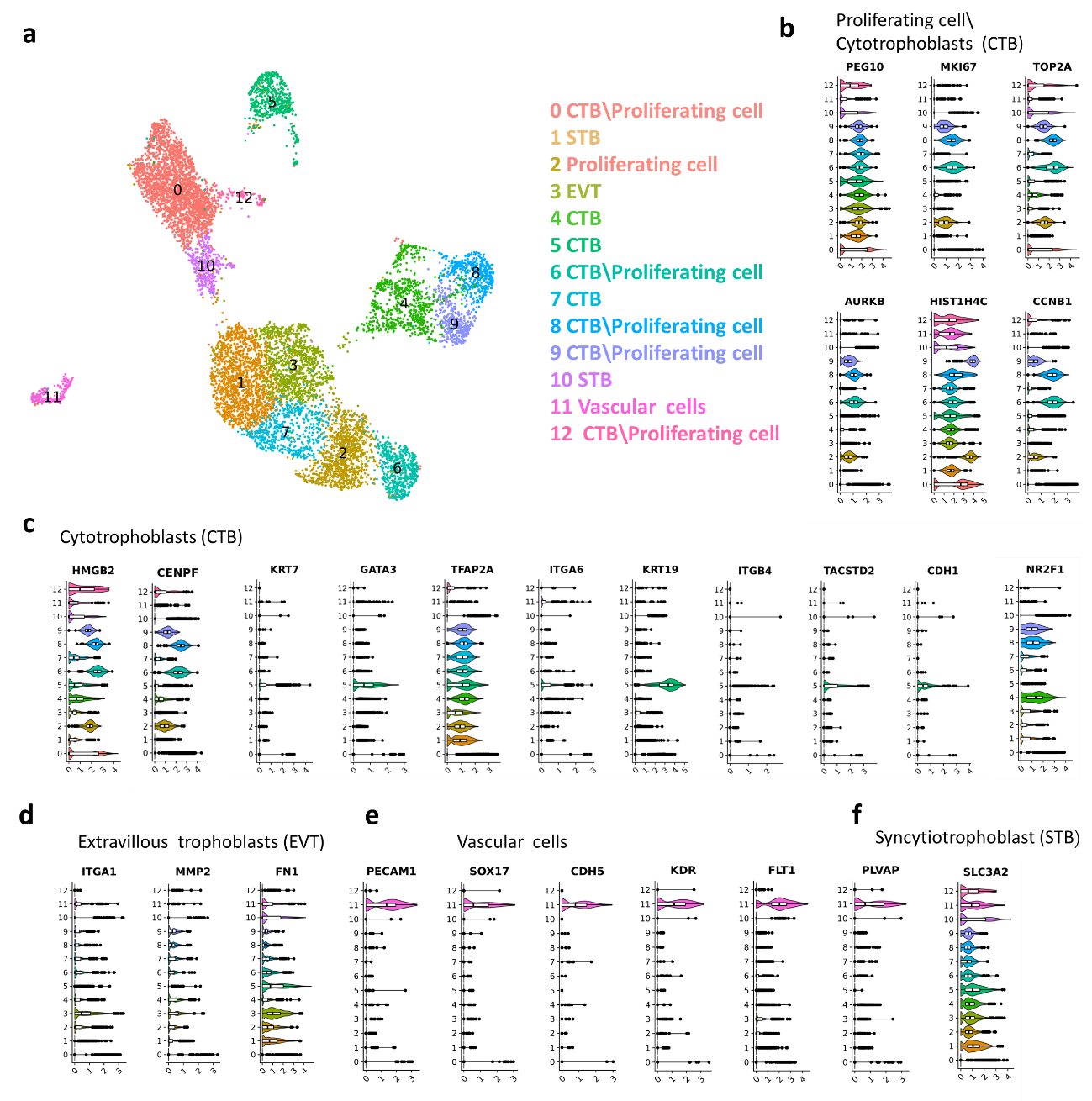


**Figure S5. Single-cell transcriptome atlas of placenta-like tissue at D24.** **a**, UMAP plot displaying 9804 cells from placenta-like organoids on day 24 of differentiation. Unsupervised clustering identified 13 clusters, which are marked by different colors. The 13 clusters were classified into five cell types based on the expression of cell-specific markers. **b-f**, Violin plots showing the expression of specific genes among the different cell types in the placenta-like tissue: proliferating cells, CTBs, STBs, EVTs, vascular cells, and STBs.


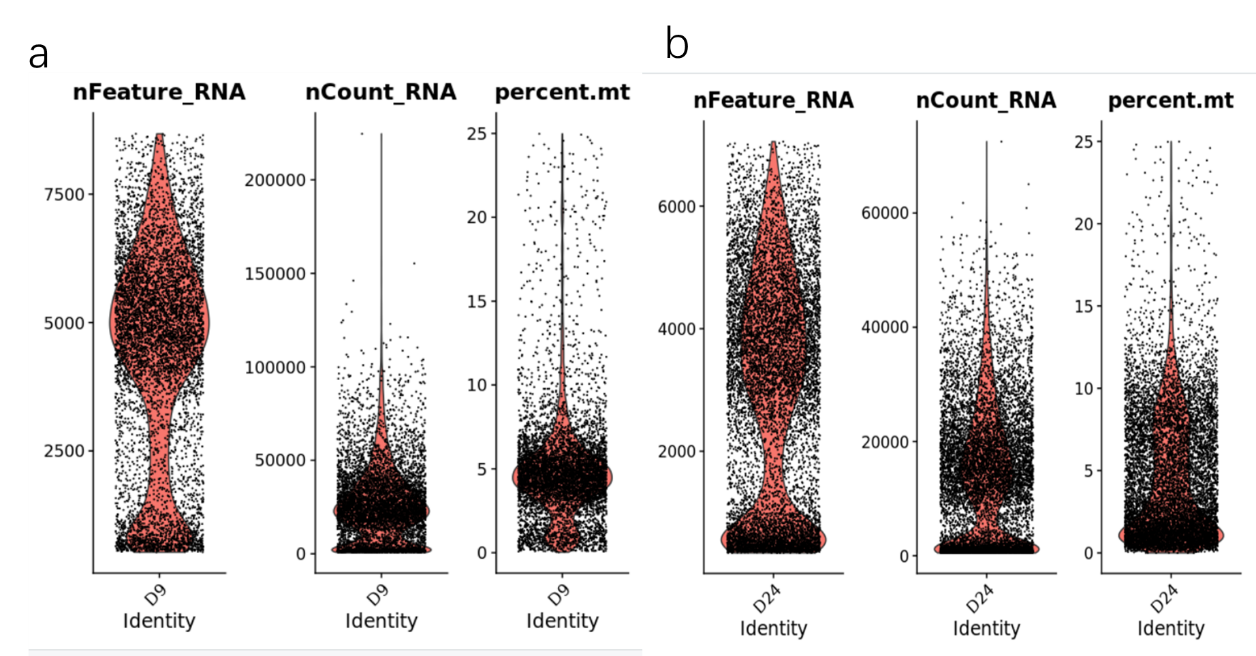


**Figure S6. Quality control and filtering of the 3D culture single-cell transcriptome atlas at day 9 and day 24.**

Table S1. List of chemical factors used to generate placenta-like tissues.

| Product | Company | Catalog No. | Final concentration |
| --- | --- | --- | --- |
| BMP4 | Gibco | PHC9531 | 10 ng/ml |
| EGF | PeproTech | AF-100-15 | 50 ng/ml |
| R-spondin 1 | R&D systems | 4645-RS-025 | 80 ng/ml |
| FGF2 | PeproTech | AF-100-18B | 100 ng/ml |
| HGF | PeproTech | 100-39 | 50 ng/ml |
| CHIR99021 | Selleckchem | S1263 | 1.5 µM |
| Y-27632 | Selleckchem | S1049 | 2 µM |
| A83-01 | Sigma | SML0788 | 500 nM |
| PGE2 | Sigma | P0409 | 2.5 µM |
| VEGFA | Biolegend | 583208 | 100 ng/ml |

Table S2. List of primary antibodies used in immunofluorescence staining (IF) and flow cytometry (FC).

| Protein | Species | Application | Catalog No. | Company |
| --- | --- | --- | --- | --- |
| HLA-G | Mouse | 1:100 (FC) | ab24384 | Abcam |
| PE-IgG1 | Mouse | 1:100 (FC) | 559320 | BD Pharmingen |
| PE-ITGA2 | Mouse | 1:100 (FC) | 555669 | BD Pharmingen |
| PE-IgG2a | Mouse | 1:100 (FC) | 555574 | BD Pharmingen |
| KDR | Mouse | 1:100 (FC) | 359915 | BD Pharmingen |
| PDGFRβ | Mouse | 1:5 (FC) | 558821 | BD Pharmingen |
| CD31 | Mouse | 1:100 (FC) | 558094 | BD Pharmingen™ |
| ENDOU | Rabbit | 1:500 (IF) | HPA012388 | Sigma |
| P63 | Mouse | 1:100 (IF) | ab735 | Abcam |
| KRT7 | Mouse | 1:100 (IF) | MA5-11986 | ThermoFisher Scientific |
| Ki67 | Rabbit | 1:500 (IF) | ab92742 | Abcam |
| CDX2 | Rabbit | 1:200 (IF) | ab76541 | Abcam |
| OCT4A | Rabbit | 1:500 (IF) | 2890 | Cell Signaling Technologies |
| GATA3 | Mouse | 1:100 (IF) | sc-268 | Santa Cruz Biotechnology |
| CGA | Rabbit | 1:100 (IF) | ab15160 | Abcam |
| E-cad | Mouse | 1:100 (IF) | 14472S | Cell Signaling Technology |
| HLA-G | Mouse | 1:500 (IF) | sc-21799 | Santa Cruz Biotechnology |
| VE-Cadherin | Rabbit | 1:400 | 89426S | CST |
| CD31 | Mouse | 1:500 (IF) | 3528S | CST |
| ColIV | Goat | 1:20(IF) | AB769 | Millipore |
| PDGFRβ | Rabbit | 1:300(IF) | 3169S | CST |
| αSMA | Goat | 1:20(IF) | AB769 | Millipore |
| ICAM1 | Rabbit | 1:400(IF) | HPA002126 | Atlas |
| vWF | Rabbit | 1:400(IF) | 65707T | CST |
| GCM1 | Rabbit | 1:200(IF) | HPA011343 | Sigma |

Table S3. Primers pairs used to detect the mRNA expression

| **Primer name** | **Sequence** |
| --- | --- |
| P63 | Fw 5′-CTG GAA AAC AAT GCC CAG A-3′  Rv 5′-AGA GAG CAT CGA AGG TGG AG-3′ |
| BMP2 | Fw 5′-CTA TCA GGA CAT GGT TGT GGA G -3′  Rv 5′-GGG AAA TAT TAA AGT GTC AAC TGG G -3′ |
| KRT17 | Fw 5′-AAG ATC CGT GAC TGG TAC CAG AGG-3′  Rv 5′-GAT GTC GGC CTC CAC ACT CAG G-3′ |
| MSX2 | Fw 5′-CGG TCA AGT CGG AAA ATT CAG -3′  Rv 5′-GGA TGT GGT AAA GGG CGT G -3′ |
| CGA | Fw 5′-ACATCCTGCAAAAAGCCCAGAGAAA-3′  Rv 5′-ACTGAAGTATTGGGGCACCCGG-3′ |
| CDX2 | Fw 5′-GAC GTG AGC ATG TAC CCT AGC-3′  Rv 5′-GCG TAG CCA TTC CAG TCC T-3′ |
| GAPDH | Fw 5′-GTG GAC CTG ACC TGC CGT CT-3′  Rv 5′-GGA GGA GTG GGT GTC GCT GT-3′ |
| TBX20 | Fw 5-′GCC AAG TAC ATA GTC CTG ATG G -3′  Rv 5′-CCG GTA AAA GGA GAA TCT GGA TG -3′ |
| CYP19A1 | Fw 5′-TGC AAA GCA CCC TAA TGT TG-3′  Rv 5′-TTT GTC CCC TTT TTC ACT GG-3′ |
